# Supplementary material for: A BioID-Derived Proximity Interactome for SARS-CoV-2 Proteins
Source: Viruses. 2022 Mar 15;14(3):611. doi: 10.3390/v14030611 (PMC8951556; doi:10.3390/v14030611)
Supplement: Supplementary file 1 [file viruses-14-00611-s001.zip › viruses-1613672-supplementary.pdf]

viruses

Article

A BioID-Derived Proximity Interactome for SARS-CoV-2 Proteins

Danielle G. May [1], Laura Martin-Sancho [2,†], Valesca Anschau [2,], Sophie Liu [3], Rachel J. Chrisopulos [1], Kelsey L. Scott [1], Charles T. Halfmann [1], Ramon Díaz Peña [2,], Dexter Pratt [3], Alexandre R. Campos [2,*] and Kyle J. Roux [1,4,*]

Supplementary Materials

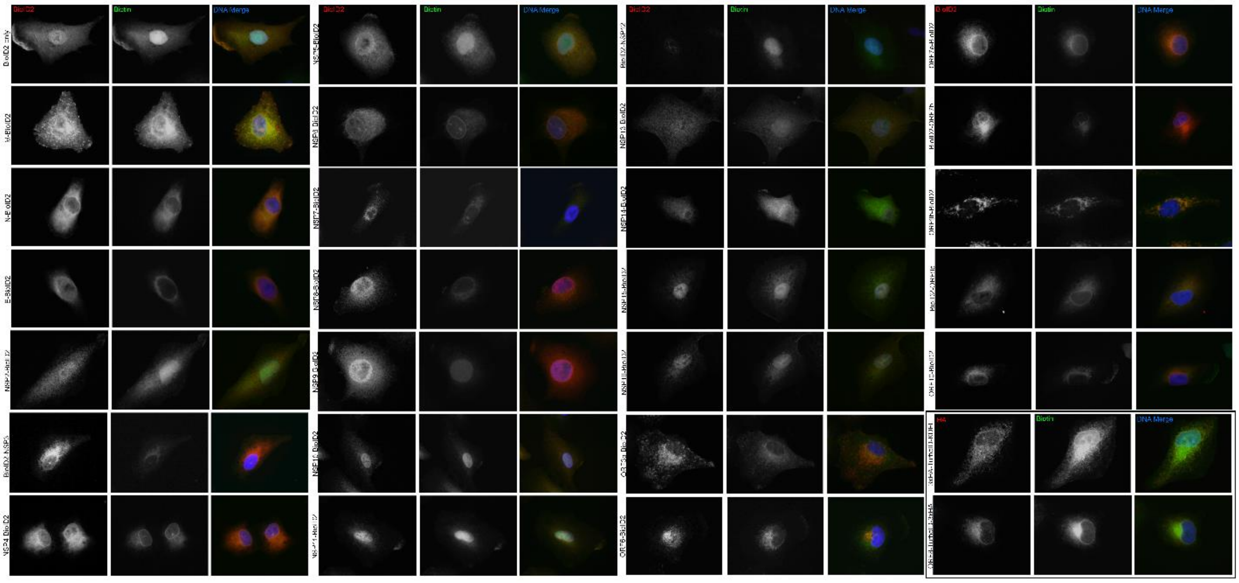

Figure S1. Epifluorescent images of all BioID fusion-protein localizations in A549 cells. A549 human lung cells stably expressing SARS-CoV-2 BioID2 fusion-proteins were assessed for fusion-protein expression and localization (red) and promiscuous biotinylation (green) following the addition of exogenous biotin.

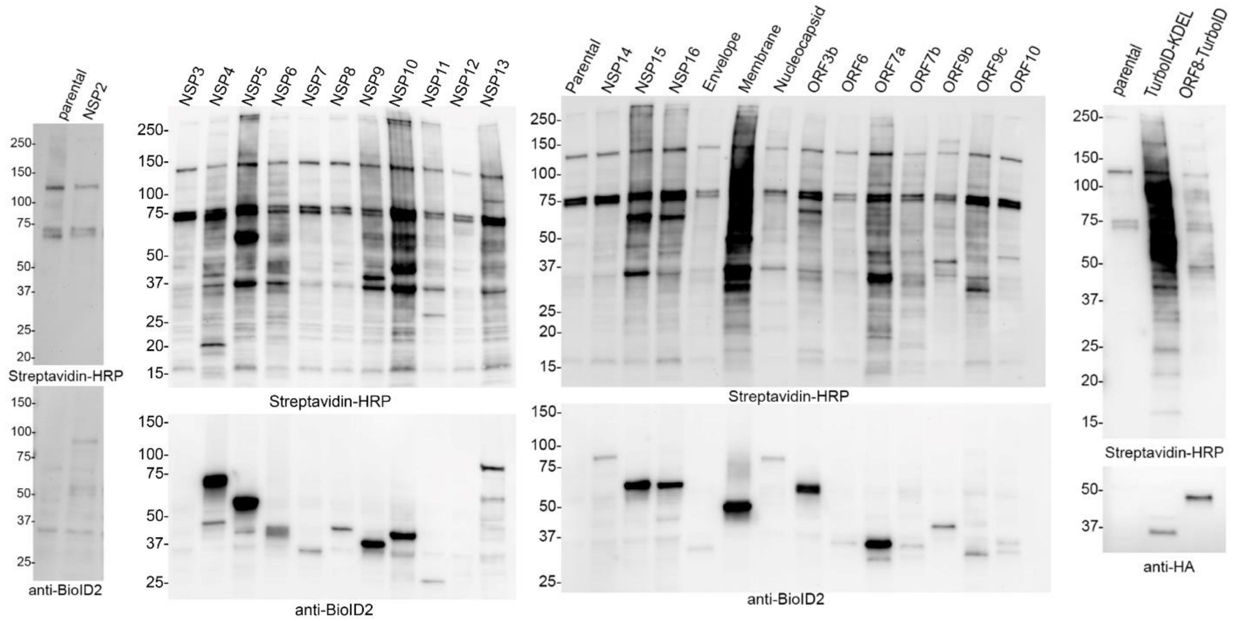

Figure S2. Western blot analysis of A549 cell stably expressing SARS-CoV-2 BioID fusion proteins. A549 cells stably expressing BioID2 fusion-proteins were evaluated for fusion-protein expression (anti-BioID2) and promiscuous biotinylation (streptavidin-HRP) and A549 cells stably expressing TurboID-KDEL control or ORF8-TurboID were evaluated for fusion-protein expression (anti-HA) and promiscuous biotinylation (streptavidin-HRP).
